# Experimental pharmacological approaches to reverse impaired awareness of hypoglycemia—a review

**DOI:** 10.3389/fphar.2024.1349004

**Published:** 2024-01-23

**Authors:** Hiba Z. Hashmi, Ameer Khowaja, Amir Moheet

**Affiliations:** ^1^ Division of Diabetes, Endocrinology and Metabolism, Department of Medicine, University of Minnesota, Minneapolis, MN, United States; ^2^ Northeast Endocrinology Associates, San Antonio, TX, United States

**Keywords:** IAH (impaired awareness of hypoglycaemia), hypoglycemia, diabetes, counterregulatory hormonal response, pharmacological

## Abstract

The colossal global burden of diabetes management is compounded by the serious complication of hypoglycemia. Protective physiologic hormonal and neurogenic counterregulatory responses to hypoglycemia are essential to preserve glucose homeostasis and avert serious morbidity. With recurrent exposure to hypoglycemic episodes over time, these counterregulatory responses to hypoglycemia can diminish, resulting in an impaired awareness of hypoglycemia (IAH). IAH is characterized by sudden neuroglycopenia rather than preceding cautionary autonomic symptoms. IAH increases the risk of subsequent sudden and severe hypoglycemic episodes in patients with diabetes. The postulated causative mechanisms behind IAH are complex and varied. It is therefore challenging to identify a single effective therapeutic strategy. In this review, we closely examine the efficacy and feasibility of a myriad of pharmaceutical interventions in preventing and treating IAH as described in clinical and preclinical studies. Pharmaceutical agents outlined include N-acetyl cysteine, GABA A receptor blockers, opioid receptor antagonists, AMP activated protein kinase agonists, potassium channel openers, dehydroepiandrosterone, metoclopramide, antiadrenergic agents, antidiabetic agents and glucagon.

## Hypoglycemia in diabetes

The global prevalence of diabetes mellitus is estimated to be around 420 million (WHO, 2023) whereas 8.4 million have type 1 diabetes (T1D) (Gregory et al., 2022). Around 72 million individuals with diabetes mellitus worldwide rely on insulin for optimal glycemic control (WHO, 2023). Hypoglycemia is considered a limiting factor for optimal management of diabetes in individuals who are on insulin therapy. The incidence of symptomatic hypoglycemia is estimated to be one to two episodes per week per person in T1D, whereas in individuals with type 2 diabetes (T2D) using insulin or sulfonylureas, it is estimated to be 0.4 episodes per week per person (Emral et al., 2017). In one study, 14% of individuals with T1D and 9% with T2D reported experiencing a severe hypoglycemia event over a period of 4 weeks (Khunti et al., 2016). Risk factors of hypoglycemia, in addition to the use of insulin, include advancing age, duration of diabetes, kidney disease, alcohol use, malnutrition, prior history of hypoglycemia and presence of impaired awareness of hypoglycemia (IAH) (Davis et al., 2010; Seaquist et al., 2013; Karter et al., 2017; Henriksen et al., 2018).

Hypoglycemia is defined as low plasma glucose that causes physiological and/or clinical impairment of patients’ wellbeing or increases risk of harm (Seaquist et al., 2013). American Diabetes Association (ADA) classifies hypoglycemia as level 1 when the glucose level is between 70 mg/dL and ≥54 mg/dL. Glucose level <54 mg/dL triggers neuroglycopenic symptoms that warrants urgent corrective intervention and is established as level 2 hypoglycemia. Lastly, level 3 hypoglycemia is defined as a severe event characterized by altered mental and/or physical status requiring assistance for the treatment of hypoglycemia (Heller, 2017).

There are several counterregulatory mechanisms that get triggered in response to lowering glucose levels. These mechanisms have evolved to preserve consistent glucose supply to the brain (Cryer, 2007). These measures can be broadly categorized as either hormonal or neurogenic with studies demonstrating an intricate interplay between these categories (Palani et al., 2023). The human body is strategically equipped with glucose sensing neurons both in central and peripheral nervous systems. It includes the oral cavity, gastrointestinal tract, portal mesenteric vein and carotid body (Donovan, 2002; Roper, 2007; Nurse, 2009; Raybould, 2010). Changes in electrochemical activity within these neurons in response to lowering glucose lead to changes in centrally located neurons in the ventromedial nucleus of the hypothalamus (VMH), tuberal nucleus, arcuate nucleus, amygdala, nucleus of the solitary tract, dorsal motor nucleus of the vagus and area postrema (Levin et al., 2006). This in turn causes reduction in insulin and increase in glucagon secretion from β and α islet cells in the pancreas respectively (Bloom and Edwards, 1975). Moreover, changes in β and α cells’ activities are also the result of direct sensing of lowering glucose levels by these cells due to the presence of glucokinase and adenosine triphosphate sensitive potassium (K-ATP) channels. Furthermore, increase in sympathetic activity leads to increased epinephrine and norepinephrine levels from the adrenal medulla (Sprague and María Arbeláez, 2011) whereas cortisol and growth hormone are thought to play counterregulatory roles in protracted hypoglycemia (Kittah and Vella, 2017). Such shifts in hormonal balance lead to decrease in glucose utilization by peripheral organs including liver, adipose tissues and musculoskeletal system, increase in hepatic and renal glycogenolysis and gluconeogenesis and increase in lipolysis thus providing glycerol and free fatty acids which serve as substrates in gluconeogenesis.

## Impaired awareness of hypoglycemia

Repeated exposure to antecedent hypoglycemia blunts the counterregulatory and symptoms responses to subsequent hypoglycemic episodes (Briscoe and Davis, 2006) leading to development of IAH. IAH is characterized by a diminished capacity to recognize the onset of low blood sugar. People with diabetes with IAH develop neuroglycopenic symptoms, that is, altered mental status before the occurrence of autonomic symptoms which include tremors, palpitations, anxiety and diaphoresis (Martín-Timón and Cañizo-Gómez, 2015). It has been estimated that 20%–25% of people with T1D and 10% with T2D have IAH (McNeilly and McCrimmon, 2018; Van Meijel et al., 2020; Li et al., 2023) which increases the risk of subsequent severe hypoglycemia (Schopman et al., 2010; Li et al., 2023). Clinical predictors associated with increased risk of IAH include longer duration of diabetes, recent history of recurrent hypoglycemia, older age and intensive diabetes regimen (Cryer, 2008). Genetic predisposition has also been associated with altered risk of IAH among people with T1D. In a study by Schouwenberg BJ et al. IAH among T1D with homozygosity for Gly16 variants of ADRB2 gene was 3.4 fold higher compared with other variants (Schouwenberg et al., 2008).

The underlying mechanisms behind development of IAH are not fully understood. There have been several postulated mechanisms explaining the relationship between recurrent hypoglycemia and IAH. The proposed mechanisms encompass changes in neurotransmission and adaptations in energy metabolism, both contributing to alterations in the brain’s ability to sense glucose. These mechanisms include: 1) Increased glucose uptake across the blood–brain barrier, enabling neurons to sustain metabolism during hypoglycemic stress; 2) Utilization of non-glucose substrates by the brain, such as lactate and ketones, to maintain energy metabolism during hypoglycemia; 3) Elevation in astrocyte glycogen levels post-hypoglycemia (supercompensation), providing additional fuel during subsequent hypoglycemic episodes; 4) Alteration in hypothalamic neurosignaling, involving increased γ amino butyric acid (GABA) signaling and diminished adenosine monophosphate kinase (AMPK) activity; 5) Increased cerebral oxidative stress; 6) Involvement of systemic mediators like endogenous opiates, adrenal neuropeptide Y, and cortisol levels. For a more detailed exploration of these mechanisms, please refer to previously published reviews (Cryer, 2013; McNeilly and McCrimmon, 2018; Stanley et al., 2019).

IAH can be partially restored by strict avoidance of hypoglycemia. However, this can be difficult to achieve and maintain long-term and could risk worsening hyperglycemia in the clinical setting (Dagogo-Jack et al., 1994; Fanelli et al., 1994; Leelarathna et al., 2013). Non-pharmacological clinical therapies including education, technology and transplantation have been eloquently explored in a recent review by Macon et al. (Macon et al., 2023) This review offers a focused review of studies investigating various pharmacological strategies aimed at preventing or reversing IAH.

## Pharmacological interventions to prevent or reverse impaired awareness of hypoglycemia

### N-acetyl cysteine

N-acetyl cysteine (NAC), provides a rich source of glutathione which is a powerful antioxidant (De Vries and De Flora, 1993). NAC administration counters reactive oxygen species (ROS) which are free radicals known to damage cellular integrity (De Vries and De Flora, 1993). NAC is commonly and effectively used in the treatment of acetaminophen poisoning for hepatocellular protection (Keays et al., 1991). It has also been studied in the treatment of pulmonary disease (Raghu et al., 2021), psychiatric illness (Berk et al., 2008) and HIV (Roederer et al., 1992) with conflicting results.

The role of NAC in preventing an impaired counterregulatory response (CRR) to recurrent hypoglycemia was initially studied by Fioramonti et al. (Fioramonti et al., 2013) in a healthy rat model. NAC administration during antecedent hypoglycemia resulted in decreased ROS production in the VMH and prevented attenuation of the CRR to subsequent hypoglycemia (Fioramonti et al., 2013). In contrast, NAC pretreatment did not prevent hypoglycemia mediated autonomic failure in the setting of prior diabetic hyperglycemia (Zhou and Routh, 2018). It was postulated that in a diabetic state, the NAC-glutathione antioxidant system may be overwhelmed by excess ROS production so targeting an additional antioxidant system may be of value (Sengupta and Holmgren, 2012).

Given the preclinical potential of NAC therapy, Moheet et al. explored its use in a randomized double blind crossover study in humans (Moheet et al., 2020). Healthy participants without diabetes were pre-treated with a 60-min 150 mg/kg NAC infusion followed by a 4-h 50 mg/kg NAC infusion or saline starting 30 min prior to the first experimental hypoglycemia clamp. Participants underwent two 2-h experimental hypoglycemic clamps on day 1 and a third hypoglycemic clamp on day 2. No significant difference in the counterregulatory hormonal or symptom response was demonstrated after NAC exposure compared to saline (Moheet et al., 2020). Thus, the initial promise of NAC therapy observed in preclinical non-diabetic rats, remained absent in non-diabetic human trials. Further exploration of its role in patients with diabetes is likely superfluous.

### GABA_A_ receptor blockers

GABA is an inhibitory neurotransmitter derived from glutamic acid decarboxylase (GAD) expressed throughout the central nervous system. Research in rodent models has indicated that hypothalamic GABA signaling plays a crucial role in regulating the CRR to hypoglycemia (Chan et al., 2006). Reduction in local glucose availability within the VMH is believed to reduce local GABA release, thereby influencing the magnitude of CRR to hypoglycemia (Zhu et al., 2010). The concentration of GABA, as measured by microdialysis, in VMH extracellular fluid has been shown to be higher and did not significantly decrease with the onset of acute hypoglycemia in rats subjected to insulin mediated recurrent hypoglycemia as compared to normoglycemic controls (Chan et al., 2008). Elevated GABAergic activity within the VMH was associated with the development of impaired CRR to hypoglycemia (Zhu et al., 2010; Chan et al., 2011). However, VMH administration of bicuculline methiodide, a GABA_A_ receptor antagonist, restored glucagon and epinephrine response to hypoglycemia ^44 45^, suggesting a possible clinical benefit of GABA antagonists as a therapeutic tool to treat IAH.

There are only a handful of studies on the influence of GABA_A_ in hypoglycemia induced counterregulatory mechanisms in humans. Blunting of CRR to hypoglycemia have been reported with the use of alprazolam, a GABA_A_ agonist (Breier et al., 1992; Giordano et al., 2003; Hedrington et al., 2010). Hedrington et al. reported that antecedent GABA_A_ agonist exposure to alprazolam resulted in blunting of autonomic and neuroendocrine CRR to hypoglycemia in healthy individuals (Hedrington et al., 2010). Consequently, pharmaceutical agents with inherent GABA *ant*agonistic properties like modafinil, which is primarily used to treat narcolepsy, gained candidacy as a therapeutic option to treat IAH. Smith et al. conducted hypoglycemic hyperinsulinemic clamp studies in nine healthy male subjects after administering 100 mg modafinil vs. placebo (Smith et al., 2004). In response to hypoglycemia, the modafinil group had modest but significantly higher autonomic symptom scores, and reduced deterioration in performance on the Stroop color word test and reaction task accuracy as compared to placebo. However, no changes in measured counterregulatory hormone levels were observed (Smith et al., 2004). Mechanisms by which modafinil exerts these effects are under study, with one study suggesting recurrent hypoglycemia impairs glucose sensitivity of perifornical hypothalamus (PFH) orexin glucose inhibited (GI) neurons which may play a role in development of IAH. In this rodent model, modafinil normalized glucose sensitivity to PFH orexin GI neurons post recurrent hypoglycemia with restoration of IAH (Patel et al., 2023). In 2021, Espes et al. published results from their proof-of-concept trial of six patients with T1D who were given a controlled release formulation of GABA, Remygen (Diamyd Medical) (Espes et al., 2021). Although primarily pursued to assess the GABA formulation for safety, hypoglycemic clamp studies during the trial demonstrated increased CRR of glucagon, epinephrine, growth hormone and cortisol (Espes et al., 2021). While not powered sufficiently for generalizable conclusions, these results were in stark contrast to prior studies supporting GABA antagonist therapy. One potential theory is that GABA has poor CNS permeability as compared to benzodiazepines, thus it primarily exerts its counterregulatory hormonal effects peripherally such as at the level of the adrenal gland (Harada et al., 2016) and pancreas (Choat et al., 2019). A recent *in vivo* protocol involving proton magnetic resonance spectroscopy to visualize GABA within the human hypothalamus during hypoglycemia can facilitate future studies to explore the exact mechanism by which GABA acts within the CNS (Park et al., 2021). There is a compelling need for large scale clinical trials exploring the role of GABA agonists and antagonists in preventing and treating IAH.

### Opioid receptor antagonists

Opioid receptor antagonists have been examined as a potential therapy to address IAH in diabetes. Endogenous opiates were shown to modulate CRR to acute hypoglycemia (McCrimmon, 2011). Intravenous administration of naloxone, an opioid receptor antagonist, enhances the CRR to acute hypoglycemia in both dogs and humans (El-Tayeb et al., 1986; Caprio et al., 1991). The exact mechanism through which endogenous opiates might impair the CRR to hypoglycemia is unknown. Opiate receptors are expressed in the VMH and activation of opioid signaling in the brain has been implicated in development of IAH (McCrimmon, 2011). Intravenous naloxone, when infused during antecedent hypoglycemia, prevented the development of defective CRR to subsequent hypoglycemia in healthy individuals and those with T1D (Leu et al., 2009; Vele et al., 2011). However, 4-week oral administration of naltrexone, another opioid receptor antagonist, did not show significant effects on hypoglycemia frequency in real-life situations or on symptoms and CRR during experimental hypoglycemia in patients with T1D and IAH (Moheet et al., 2015). In another study (Naik et al., 2017), overnight administration of two doses of oral naltrexone, showed modest increase in plasma epinephrine during hypoglycemia compared to placebo without changes in other counterregulatory hormones.

### AMP-activated protein kinase agonists

AMPK is a serine-threonine kinase which is responsible for phosphorylating several proteins involved in cell metabolism (Garcia and Shaw, 2017). Given most neurons do not possess energy stores; neuronal AMPK can act as a glucose sensor. The role of AMPK in regulating CRR to hypoglycemia has been investigated in animal studies. McCrimmon et al. injected agent 5-aminoimidazole-4-carboxamide ribonucleotide (AICAR) into the VMH of rats to stimulate AMPK during hyperinsulinemic-hypoglycemic clamp studies (McCrimmon et al., 2004). While no change in hormonal counterregulation was observed; there was up to fourfold increase in hepatic glucose production as compared to controls. McCrimmon et al. repeated this study in rats with antecedent recurrent hypoglycemia and reported a significant improvement in the CRR (epinephrine and glucagon) in addition to increased hepatic glucose production (McCrimmon et al., 2006). Alquier et al. also demonstrated that intracerebrovascular injection of AICAR to stimulate AMPK in a rat model of repetitive neuroglycopenia partially restored the CRR (Alquier et al., 2007). Fan et al. examined AMPK activation in a rat model of T1D (Fan et al., 2009). Four rat groups were studied, two subjected to chronic hyperglycemia and two to recurrent hypoglycemia for 3 days. This was followed by a hyperinsulinemic hypoglycemic clamp study in all four groups with concurrent AICAR vs. saline injection into the VMH. They noted a significantly improved glucagon CRR to hypoglycemia in the AICAR treated diabetic rats as compared to saline treated controls indicating that AMPK manipulation could be a useful pharmaceutical tool to treat IAH (Fan et al., 2009).

Given AICAR requires intracerebral injection, the need for a peripherally administered AMPK activator with central nervous system (CNS) permeability was identified. Metformin is known to activate AMPK and has recently been found to exert neuroprotective effects by crossing the blood brain barrier (Sharma et al., 2021; Sharma et al., 2023). The role of metformin in treating IAH requires further study. In 2021, Cruz et al. did study a novel, brain permeable compound R481, which is more potent than metformin, in a healthy rat model. They demonstrated that this metformin-like compound amplified peak plasma glucagon levels in response to hypoglycemia in healthy rats (Cruz et al., 2021). The efficacy and safety of R48 in preventing IAH in a diabetic rodent population and in humans requires further exploration.

### Potassium channel openers (KCO)

ATP sensitive potassium channels notably act as glucose sensors in the pancreas for hormonal insulin mediated glycemic regulation. ATP sensitive potassium channels comprise two subunits, Kir6.2 and SUR-1 (Seino and Miki, 2003) and have also been identified within the glucose sensing VMH region (Kang et al., 2004). McCrimmon et al. injected the KCOs diazoxide and NN414 into the VMH of healthy rats with significant amplification of epinephrine and glucagon CRR to hypoglycemia. This effect was maintained in diazoxide treated rats subjected to antecedent recurrent hypoglycemia, suggesting a possible therapeutic role for KCOs with an existing impaired CRR to hypoglycemia (McCrimmon et al., 2005). However, there is evidence that prolonged exposure to KCOs like NN414 may result in a conformational change in the ATP sensitive potassium channel, resulting in attenuated glucose sensing over time (Haythorne et al., 2016).

In a human trial of patients with long-standing T1D, diazoxide at a dose of 7 mg/kg was shown to significantly upregulate the catecholaminergic CRR (George et al., 2015). Moreover, E23K, a genetic polymorphism of the potassium channel, was identified which predicted a blunted response to diazoxide therapy (George et al., 2015). There was no appreciable symptom score response, however this may have been a result of the small sample size. Future large scale clinical trials should investigate the impact of prolonged diazoxide therapy as well as the clinical utility of genotype directed stratification of participants (George et al., 2015; Farrell and McCrimmon, 2021).

### Dehydroepiandrosterone

Dehydroepiandrosterone (DHEA) and its sulfated metabolite form is a steroid hormone. It is considered to play a role in CRR to hypoglycemia, given its inherent stimulation of N-methyl-D-aspartate and nitric oxide synthase along with GABA and glucocorticoid antagonism (Mikeladze et al., 2016). Mikeladze et al. administered oral DHEA or placebo as healthy humans underwent a standard model of repeated experimental hypoglycemia to induce IAH. High doses of oral DHEA given prior to hypoglycemia clamps preserved the hypoglycemia related hormonal, autonomic and symptom CRR (Mikeladze et al., 2016). Future studies in patients with diabetes and at submaximal doses will be needed to establish its clinical efficacy in treating IAH.

### Metoclopramide

Metoclopramide has antiemetic properties and is commonly used in the management of diabetic gastroparesis and gastroesophageal reflux disease. It antagonizes dopamine D2 receptors peripherally as well as within the CNS. In rodent models of IAH, metoclopramide has shown promising results as a potential therapeutic agent to prevent or restore CRR to hypoglycemia (VIEIRA DE ABREU et al., 2018; DEVORE et al., 2022). There is an ongoing randomized clinical trial (Clinical Trials.gov ID NCT03970720) examining the role of metoclopramide in restoring hypoglycemia awareness in human subjects with T1D (ClinicalTrials, 2023b).

### Adrenergic blockade

The adrenergic response to hypoglycemia is essential in triggering sympathetic symptoms that make us aware of hypoglycemia. However, repeated activation of the adrenergic system has been postulated to contribute to development of IAH. In healthy humans, antecedent activation of adrenergic receptors through repeated epinephrine infusions, without inducing hypoglycemia, lead to impairment in CRR to subsequent hypoglycemia (Lontchi-Yimagou et al., 2020). β-adrenergic blockage has been shown to prevent the deleterious impact of antecedent hypoglycemia on CRR to subsequent hypoglycemia (Ramanathan and Cryer, 2011). In a diabetic rat model exposed to recurrent hypoglycemia, administration of the non-specific β-adrenergic receptor blocker, carvedilol, prevented the development of impaired CRR to hypoglycemia (Farhat et al., 2021). Future studies are needed to examine the impact of adrenergic blockage for prevention or treatment of IAH in humans.

### Other pharmacological agents

Glucagon-like Peptide (GLP) 1 receptor agonists and Sodium glucose cotransporter (SGLT2) inhibitors are very effective classes of medication approved for use in people with T2D. These medications may reduce the risk of hypoglycemia by improving glycemic variability. Studies with dapagliflozin, an SGLT2-inhibitor and exenatide, a GLP-1 agonist examined the impact of these agents on IAH in people with T1D by potentially reducing the risk of hypoglycemia. However, in short-term studies these agents did not improve the hormonal CRR or symptom response in people with T1D (Van Meijel et al., 2019; van Meijel et al., 2021; Boeder et al., 2023; Urakami et al., 2023). In another study, administration of a fixed dose subcutaneous continuous infusion of glucagon for 4 weeks, did not improve epinephrine response to hypoglycemia in individuals with T1D and IAH (ClinicalTrials, 2023a).

## Conclusion

The intricate web of complex pathophysiological processes contributing to impaired awareness of hypoglycemia poses a significant challenge in pinpointing a singular pharmaceutical solution for treatment. While some studies have shown promising candidates, there is a dearth of literature in humans to identify a safe pharmaceutical agent to restore hypoglycemia awareness. Considering the multitude of factors influencing cerebral hypoglycemia sensing mechanisms, it is probable that a combination of pharmaceutical approaches will be necessary. Consequently, there is an urgent need for further clinical trials in humans to address this pressing issue.
